# Recurrent Urinary Tract Infections: Unraveling the Complicated Environment of Uncomplicated rUTIs

**DOI:** 10.3389/fcimb.2021.562525

**Published:** 2021-07-22

**Authors:** Jonathan Josephs-Spaulding, Thøger Jensen Krogh, Hannah Clara Rettig, Mark Lyng, Mariam Chkonia, Silvio Waschina, Simon Graspeuntner, Jan Rupp, Jakob Møller-Jensen, Christoph Kaleta

**Affiliations:** ^1^ Research Group Medical Systems Biology, Institute of Experimental Medicine, Christian-Albrechts-Universität, Kiel, Germany; ^2^ Department of Biochemistry and Molecular Biology, University of Southern Denmark, Odense, Denmark; ^3^ Department of Infectious Diseases and Microbiology, University of Lübeck, Lübeck, Germany; ^4^ Research Group Nutriinformatics, Institute of Human Nutrition and Food Science, Christian-Albrechts-Universität, Kiel, Germany; ^5^ German Center for Infection Research (DZIF), Partner site Hamburg-Lübeck-Borstel-Riems, Lübeck, Germany

**Keywords:** recurrent urinary tract infections (rUTIs), human microbiome, UPEC, dysbiosis, microbial ecology

## Abstract

Urinary tract infections (UTIs) are frequent in humans, affecting the upper and lower urinary tract. Present diagnosis relies on the positive culture of uropathogenic bacteria from urine and clinical markers of inflammation of the urinary tract. The bladder is constantly challenged by adverse environmental stimuli which influence urinary tract physiology, contributing to a dysbiotic environment. Simultaneously, pathogens are primed by environmental stressors such as antibiotics, favoring recurrent UTIs (rUTIs), resulting in chronic illness. Due to different confounders for UTI onset, a greater understanding of the fundamental environmental mechanisms and microbial ecology of the human urinary tract is required. Such advancements could promote the tandem translation of bench and computational studies for precision treatments and clinical management of UTIs. Therefore, there is an urgent need to understand the ecological interactions of the human urogenital microbial communities which precede rUTIs. This review aims to outline the mechanistic aspects of rUTI ecology underlying dysbiosis between both the human microbiome and host physiology which predisposes humans to rUTIs. By assessing the applications of next generation and systems level methods, we also recommend novel approaches to elucidate the systemic consequences of rUTIs which requires an integrated approach for successful treatment. To this end, we will provide an outlook towards the so-called ‘uncomplicated environment of UTIs’, a holistic and systems view that applies ecological principles to define patient-specific UTIs. This perspective illustrates the need to withdraw from traditional reductionist perspectives in infection biology and instead, a move towards a systems-view revolving around patient-specific pathophysiology during UTIs.

## Introduction

Recent advances in DNA and RNA sequencing contradicts prior assumptions that human urine is sterile; instead it harbors a unique microbiome with ecological interactions in health and disease (Wolfe et al., 2012; Hilt et al., 2014; Alteri and Mobley, 2015; Whiteside et al., 2015; de Vos et al., 2017; Aragón et al., 2018). As the physiological role of the bladder is to store nutrients and waste products in the form of urine, healthcare practitioners must consider the unique metabolism of bladder-associated microbial communities within this niche (Subashchandrabose et al., 2014; Alteri and Mobley et al., 2015; Conover et al., 2016; Horsley et al., 2018; Martín-Rodríguez et al., 2020). More so, with the emergence of pathogens that are resistant to first-line antibiotics, there is a pressing need to reinterpret uncomplicated UTIs, as an ever-changing and complicated pathology in humans (Mediavilla et al., 2016; Zilberberg et al., 2017). With the development of novel systems biology approaches and next generation methods, new viewpoints of UTIs and human-associated infections are being uncovered.

Most recently, the American Urological Association estimates that 150 million UTIs occur worldwide annually and cost up to $6 billion USD in healthcare costs (Flores-Mireles et al., 2015; AUA, 2016). This has led to the misapplication of antibiotics, likely resulting in long-term effects upon the interconnected gastrointestinal tract, vagina, and general urinary system (Kostakioti et al., 2012; Bartoletti et al., 2016; Nielsen et al., 2016; Gottschick et al., 2017; Thomas-White et al., 2018). Uropathogenic *Escherichia coli* (UPEC) primarily causes UTIs and is isolated from approximately 80% of patients (Flores-Mireles et al., 2015). Other pathogens such as *Enterococcus faecalis, Klebsiella pneumoniae, or Proteus mirabilis* can also be isolated from UTI patients (Abat et al., 2015; Thänert et al., 2019). UTIs begin at the urethra, colonize the bladder, and ascend to the kidneys through a multitude of mechanisms such as evading host protective factors or inhibiting host immunoglobulin A transport (Rice et al., 2005; Ashkar et al., 2008). The primary microbial strain causing the rUTI may originate from new colonizers deriving from various environmental reservoirs such as: a sexual partner, contaminated foods, or fecal/gut contamination of the urinary tract (Scholes et al., 2000; Nordstrom et al., 2013; Foxman, 2014; Gilbert et al., 2017; Thänert et al., 2019). While strains which initiate infection may cause a rUTI relapse, it is suggested that the initial infection caused by one UPEC strain primes the bladder for a new strain (or slightly similar) within several hours, which is diagnosed by two separate cultures over a period of six months (Andersen et al., 2012; Luo et al., 2012; Schreiber et al., 2017; Anger et al., 2019). Specifically, uncomplicated UTIs are acute infections of urinary tracts without anatomical or physiological defects that would make a patient more susceptible to initial urinary tract infections (Hooton, 2012). Women are significantly at a higher risk for contracting a UTI as compared to men. Previous reports suggest that one-third of all women under the age of 26 will experience a UTI and 50% of those women will experience a subsequent UTI episode (Foxman, 2002; Brumbaugh et al., 2013).: it is estimated that (This figure has potentially increased due to the emergence of multidrug resistance (MDR) UTIs (; Hooton et al., 2004; Nordstrom et al., 2013; Zilberberg et al., 2017).

**Figure 1:**
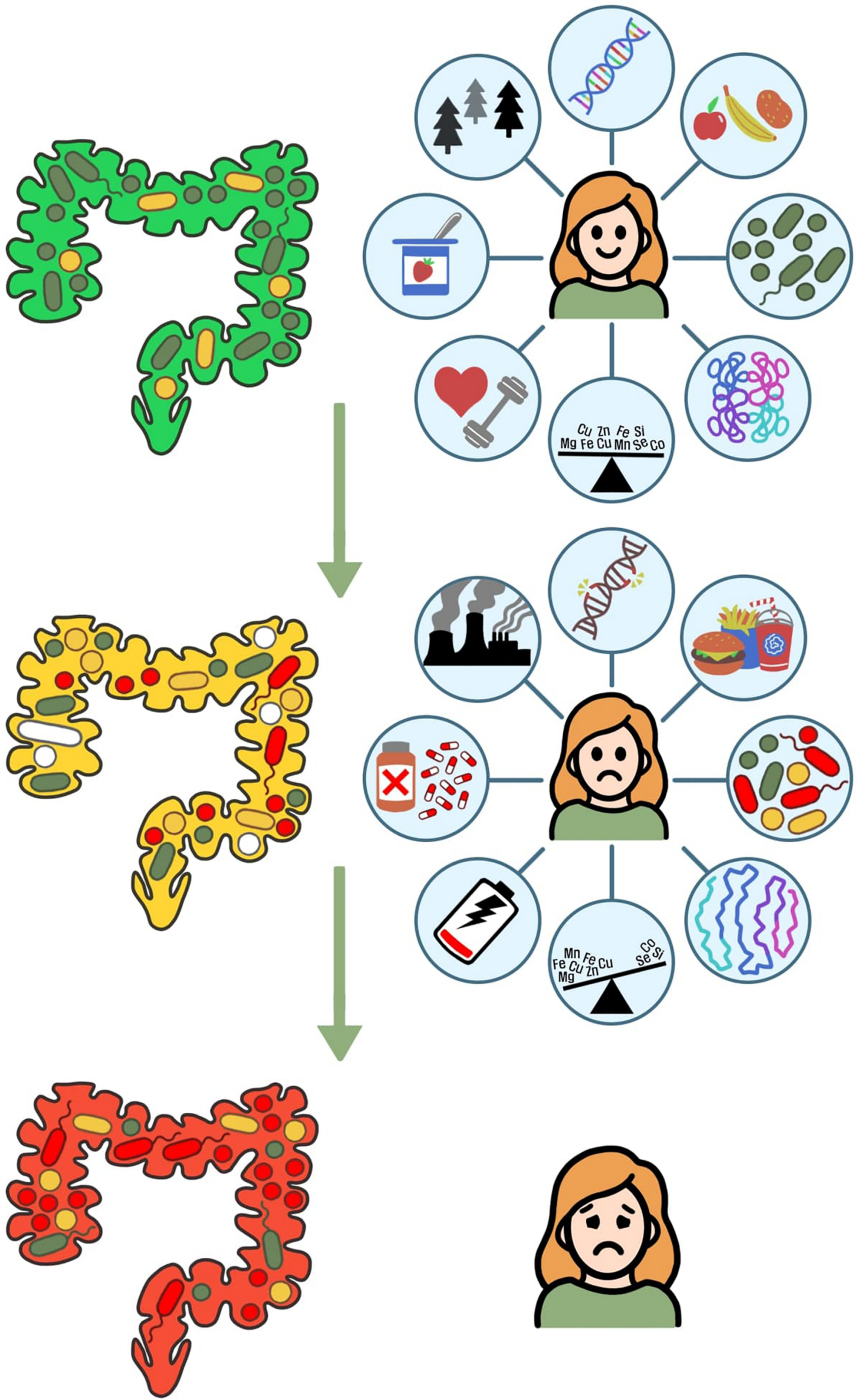
A healthy host and associated commensal microbiota enforces an environment that prevents colonization by invasive pathogens. However dysregulation of host physiology and native microbial communities is influenced by environmental factors. Through adverse community stressors such as DNA or protein impairment, poor diet, depleted microbial diversity, excessive metals, an inactive lifestyle, antibiotic usage, and adverse environment. The two-pronged attack of dysregulation of both microbial communities and host homeostasis leads to dysbiosis of beneficial bacteria, thus facilitating disease outcomes of individuals and formation of new microbial communities reflective of the pathology.

As knowledge in modeling the fine-scale and dynamic changes in biological organisms increases, the context of personalized treatment strategies must also update with observations in molecular systems biology and microbiome sciences (Thiele et al., 2013; Thiele et al., 2020). Ideally, this includes withdrawing from reductionist approaches to diagnose UTIs and moving towards models that input multiple human data sources to create patient-specific models to characterize infection in a holistic view (**Figure 1**). Overall, our understanding of both host-microbiome interactions and rUTI pathophysiology elicits an update to the present concept of infection biology by redefining infectious diseases through a modern lens.

## Microbiota of the Female Urogenital System

A culturable urinary tract microbial community exists within healthy individuals (Hilt et al., 2014). Specifically, the urobiome has been hypothesized to shift both microbial abundance and predicted metabolic pathways associated with various patient-specific urological morbidities or infections (Shoskes et al., 2016; Aragón et al., 2018). While less reports of the male urinary microbiome exist, both sexes share a similar core microbiome with genera from Lactobacillus, *Streptococcus*, and *Corynebacterium;* the latter is more common in men and is regularly associated with the skin microbiome (Fouts et al., 2012; Nelson et al., 2012; Lewis et al., 2013). Specifically, the urine of healthy females is characterized by the presence of *Corynebacterium, Lactobacillus, Staphylococcus*, and *Streptococcus* that tends to fluctuate abundance during periods of health and disease (Fouts et al., 2012; Aragón et al., 2018). Additionally when assessed by microbiome sequencing, it has been found that female urinary tract samples mainly consist of organisms from the phyla *Actinobacteria (Actinomyces* & Arthrobacter) and *Bacteroidetes (Bacteroides)*, which are typically absent from their male counter parts (Lewis et al., 2013). More so, *E.* coli is readily cultured from 91% of healthy women and only 25% of men, highlighting a stark difference in *E. coli* that is cultured as a residential bacteria from the female urobiome (Ipe et al., 2013). Generally, these differences between males and females leading to unique microbiomes could simply be due to both anatomical and hormone differences between sexes (Whiteside et al., 2015).

When assessing cohorts by age, it becomes clear aging affects normal physiology and disease types (Irizar et al., 2018). Specifically a core female urine microbiota which parallels aging and age-related morbidities was identified and is manifested as asymptomatic bacteriuria (Lewis et al., 2013). In the case of patients with so-called asymptomatic bacteriuria, *E. coli* typically acts as symbionts (Godaly et al., 2016). Therefore, unnecessary and excessive application of antibiotics to “treat” asymptomatic bacteriuria in differing age groups leads to long-term consequences by depleting the resilient urinary system’s microbiota, thus driving an increased prevalence of multidrug resistance (MDR) pathogens within the urinary tract across patients (Cai et al., 2015; Ipe et al., 2016; Zilberberg et al., 2017). This suggests that the definition of UTI diagnosis: ‘the detection of a pathogen from the not be sterile during all periods of health. **Table 1** summarizes the known microbial diversity of urine of a symptomatic patient’, must be modified in acknowledgement that human urine may is both the male and female urogenital system during periods of health, disease, and the asymptomatic colonization of microbiota.

**Table 1:** Comparison of known microbial diversity of human male and female urogenital systems during the onset of physiological disequilibrium.

Male	Female	Common to Male & Female	Location	Citation
Actinobaculum,	Actinobaculum,	Actinobaculum Aerococcus Atopobium Azospira Butyricicoccus Campylobacter Catonella Corynebacterium Dialister Finegoldia Fusobacterium Gardnerella Lactobacillus Mobiluncus Murdochiella Peptococcus Peptoniphilus Peptostreptococcus Porphyromonas Prevotella Proteiniphilum Saccharofermentans Sneathia Soehngenia Staphylococcus	Asymptomatic Urine	Lewis et al. (2013)
Aminobacterium,	Actinomyces,			
Anaerococcus,	Aerococcus,			
Anaerophaga,	Anaerococcus,			
Anaerosphaera,	Anaerosphaera,			
Aerococcus,	Anaerovorax,			
Anaerotruncus,	Arcanobacterium,			
Atopobium,	Arthrobacter,			
Atopostipes,	Atopobium,			
Azospira,	Azospira,			
Butyricicoccus,	Brevibacterium,			
Campylobacter,	Brooklawnia,			
Catonella,	Butyricicoccus,			
Corynebacterium,	Campylobacter,			
Dialister,	Catonella,			
Eubacterium,	Caulobacter,			
Filifactor,	Coriobacterium,			
Finegoldia,	Corynebacterium,			
Fusobacterium,	Dialister,			
Gardnerella,	Enterobacter,			
Gemella,	Enterobacter,			
Gordonibacter,	Enterococcus,			
Kocuria,	Facklamia,			
Lactobacillus,	Fastidiosipila,			
Lactonifactor,	Finegoldia,			
Marixanthomonas,	Flavonifractor,			
Megasphaera,	Friedmanniella,			
Microvirgula,	Fusobacterium,			
Mobiluncus,	Gallicola,			
Murdochiella,	Gardnerella,			
Mycoplasma,	Gulosibacter,			
Peptococcus,	Helcococcus,			
Peptoniphilus,	Howardella,			
Peptostreptococcus,	Incertae Sedis,			
Porphyromonas,	Jonquetella,			
Prevotella,	Lachnospiracea,			
Pseudomonas,	Lactobacillus,			
Rikenella,	Methylovirgula,			
Proteiniphilum,	Microvirgula,			
Pseudoramibacter,	Mobiluncus,			
Saccharofermentans,	Modestobacter,			
Sediminitomix,	Murdochiella,			
Sneathia,	Negativicoccus,			
Soehngenia,	Neisseria,			
Staphylococcus,	Oligella,			
	Paraprevotella,			
	Parvimonas,			
	Pelomonas,			
	Peptococcus,			
	Peptoniphilus,			
	Peptostreptococcus,			
	Porphyromonas,			
	Prevotella,			
	Propionimicrobium ,			
	Proteiniphilum,			
	Rhodococcus,			
	Rhodopila,			
	Saccharofermentans,			
	Sneathia,			
	Soehngenia,			
	Sporanaerobacter,			
	Staphylococcus ,			
	Stenotrophomonas,			
	Streptococcus,			
	Streptophyta,			
	Sutterella,			
	Tepidimonas,			
	Tessaracoccus,			
	Thermoleophilum ,			
	Varibaculum,			
Atopobium Corynebacterium Staphylococcus Streptococcus Veillonella	Corynebacterium, Escherichia coli Lactobacillus Prevotella Staphylococcus aureus Streptococcus (beta-hemolytic)	Corynebacterium, Staphylococcus Streptococcus	Healthy Urine	Fouts et al., 2012
	Atopobium vaginae Lactobacillus species (Lactobacillus iners, L. crispatus, L. gasseri, or L. jensenii) Leptotrichia spp. Megasphaera	N/A	Healthy Vagina	Lamont et al., 2011
N/A	Lactobacillus spp. Pseudomonas spp. Pseudomonadaceae	N/A	Non-Pregnant Urine	Yoo et al., 2016
N /A	Bacillus spp. Lactobacillus spp. Ureaplasma spp. Veillonellaceae	N /A	Pregnant Urine	Yoo et al., 2016
N /A	Atopobium spp. Fusobacterium spp. Lactobacillus spp. Megasphaera spp. Megasphaera spp. Prevotella spp. Sneathia spp. Ureaplasma spp Ureaplasma urealyticum Veillonellaceae	N /A	Preterm Delivery Urine	Yoo et al., 2016
Enterococcus faecalis Escherichia coli Klebsiella pneumoniae	Gardnerella Klebsiella oxytoca	N /A	Neuropathic Urine - Void spontaneously	Fouts et al., 2012
Escherichia coli Klebsiella pneumoniae Proteus	Escherichia coli	Escherichia coli	Neuropathic Urine - Intermittent Catheter	Fouts et al., 2012
Enterococcus faecalis Escherichia coli (ESBL) Klebsiella pneumoniae Providencia stuartii Pseudomonas aeruginosa	Citrobacter koseri (diversus) Enterococcus faecalis Escherichia coli Gram Negative Rods,	Enterococcus faecalis	Neuropathic Urine - Foley Catheter	Fouts et al., 2012
	Actinobaculum, Actinomyces, Aerococcus, Anaerococcus, Arthrobacter, Bifidobacterium spp., Campylobacter, Clostridium Corynebacterium, Enterococcus, Escherchia, Lactobacillus, Gardnerella, Prevotella, Proteus, Psuedomonas, Serratia, Staphylococcus, Streptococcus	N /A	Overactive Bladder	Hilt et al., 2014; Curtiss et al., 2017
N /A	Atopobium vaginae, Chlamydia trachomatis, Gardnerella vaginalis, Mobiluncus species, Mycoplasma hominis, Trichomonas vaginalis,	N /A	Bacterial Vaginosis	Lamont et al., 2011
Chlamydia trachomatis, Neisseria gonorrhoeae, Treponema pallidum	Atopobium vaginae Bifidobacterium, C. trachomatis, Dialister, Gardnerella vaginalis, Leptotrichia, M. genitalium, Megasphaera elsdenii, Mobiluncus spp. Mycoplasma hominis, N. gonorrhoeae, Prevotella spp.	Chlamydia trachomatis, N. gonorrhoeae,	Male Penis or Female Vagina w/ HIV Infections	Saxena et al., 2012
	Anaerococcus, Bifidobacterium, Coriobacteriaceae, Dialister, Lactobacillus, Megasphaera, Prevotella, Rhodococcus, Shuttleworthia, Streptococcus,	N /A	Interstital Cystitis / Bladder Pain (Urine)	Meriwether et al., 2019
	Bifidobacterium, Coriobacteriaceae, Dialister, Finegoldia, Lactobacillus, Megasphaera, Mobiluncus, Peptoniphilus, Prevotella, Shuttleworthia,	N /A	Interstital Cystitis / Bladder Pain (Vagina)	Meriwether et al., 2019
Candida Albicans, Enterobacter spp., Enterococcus, E. coli, Klebesilla spp. Proteus mirabilis, Pseudomonas aeurignosa	Blautia, Klebsiella, Prevotella,	Klebsiella spp	Type 2 diabetes Patients w/ Asymptomatic Bacteriuria (Urine) Bladder Cancer	Balamuruganvelu et al., 2017; Liu et al., 2017
Acinetobacter baylyi, Anaerococcus, Atopostipes, Candidatus Limnoluna, Carnobacteriaceae, Rickettsiales, Rothia, Rubrobacteria, Rubrobacterales, Sphingobacteriaceae		N /A	Bladder Cancer	Wu et al., 2018
Alloscardovia omnicolens, Anaerococcus lactolyticus, Anaerococcus murdochii, Auritidibacter ignavus Corynebacterium coyleae, Corynebacterium genitalium Corynebacterium minutissimum, Enterobacteriaceae, Gammaproteobacteria, Gardnerella vaginalis Haemophilus haemolyticus Propionimicrob um lymphophilum, Streptococcus, Ureaplasma parvum Ureaplasma urealyticum,		N /A	Prostate Cancer	Shrestha et al., 2018

N/A, Not Applicable.

### UTIs and Vaginal Microbial Communities

The dynamic exchange of microbiota, between the vagina and urinary tract illustrates a complex environment which is dependent on microbial composition. Probiotic strains of *Lactobacillus (L. crispatus, L. gasseri, L. iners, and L. jensenii)* that are the dominant bacteria of the vagina are demonstrated to repel or suppress non-native pathogens in environments through the production of secondary metabolites and control of environmental pH (Mirmonsef et al., 2014; Brubaker and Wolfe, 2017; Tachedijian et al., 2017; O’ Hanlon et al., 2019). Specifically, recent metagenomic sequencing of both the female urinary tract an vagina have highlighted highly similar microbiota between both systems such as E. coli, Lactobacillus spp. and Streptococcus anginosus suggesting a degree of cross-talk between both environments (Thomas-White et al., 2018). These findings suggest a dynamic continuous interplay between beneficial commensals and invasive pathogens (Vodstrcil et al., 2017). For example, loss or depletion of one species of Lactobacillus spp. leads to other organisms invading the previously occupied niche (van de Wijgert et al., 2014). More so, loss of commensal microbial communities consisting of probiotic strains likely leads to dysbiosis and an increased risk for UTIs, rUTIs, or infections from other bacteria and viruses (Sewankambo et al., 1997; Martin et al., 1999; Lamont et al., 2011; Stapleton et al., 2011; Gilbert et al., 2017). While microbial dysbiosis is a known mechanism underlying bacterial vaginosis, it remains unknown how dysbiosis of microbial communities impacts the urinary tract (Zozaya et al., 2016; Liu et al., 2017).

While microbiota of the host urogenital system are ultimately connected, microbiota can also be shared between the urogenital systems of two hosts.Interestingly, microbiome studies of patients with bacterial vaginosis highlighted a shared microbiome between the penile/urethral microbiota and vaginal microbiota (Zozaya et al., 2016). Specifically, penile-vaginal sex is the primary driver for the sexual exchange and increase of *G. vaginalis* in individuals with or without bacterial vaginosis (Vodstrcil et al., 2017). Furthermore, the increased presence of *G. vaginalis* in tandem with biofilm-producing communities drives an intense competition for resources, leading to a decreased presence of *Lactobacillus* spp. and an increased risk for acquiring urogenital infections (Machado et al., 2013). This loss of *Lactobacillus* spp. shifts the host vagina towards a more alkaline environment to include more diverse microbial communities such as *Anaerococcus*, *Atopobium, Bacteroides* spp. *Gardnerella, Mobiluncus* spp, *Mycoplasma, Peptoniphilus, Peptostreptococcus* spp. *Prevotella, and Streptococcus* that is found in patients with bacterial vaginosis (Lamont et al., 2011; Ravel et al., 2011; Mirmonsef et al., 2014; Onderdonk et al., 2016).

### Host Gut and Urogenital Microbiome Crosstalk Underlying rUTIs

**Figure 2:**
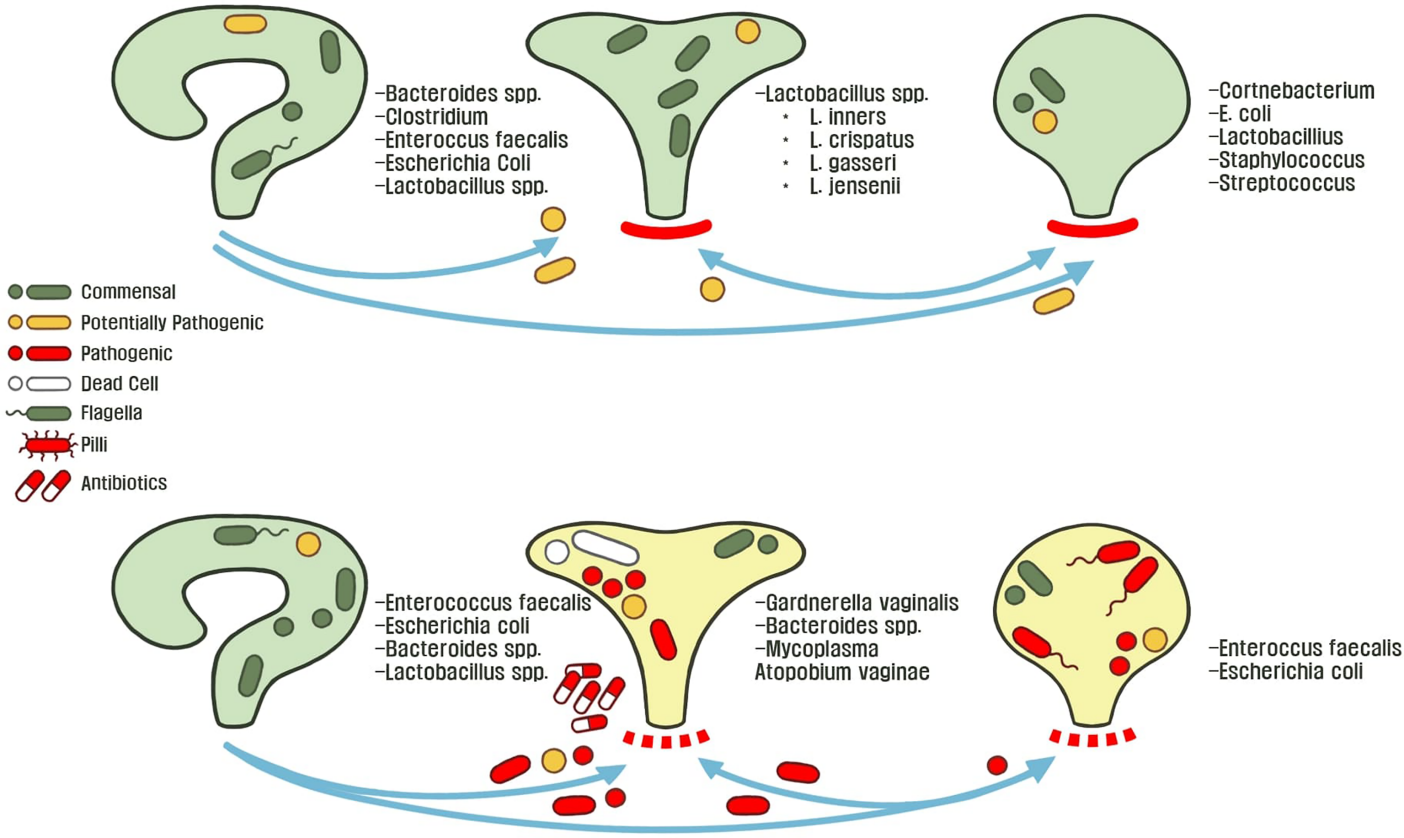
The healthy or asymptomatic gut, vagina, and bladder microbiome is stable across individuals conditions. These microbiota protect their host-associated niche from foreign pathogens by controlling abiotic factors and outcompeting potentially invasive microbiota. Microbial dysbiosis and an adverse environment disrupts host homeostasis through various mechanisms such as: poor hygiene, metabolic changes (menopause, metabolic diseases, etc.), exposure to environmental metals or antibiotics, and consumption of food-borne pathogens. This allows for microbiota to transit between the perianal-urogenital pathway and in turn shapes other microbial communities towards dysbiosis and predisposes individuals to UTI pathophysiology.

Gut microbiota play a unique role in the hypothetical fecal-perianal-urethra transmission route:fecal-associated microbiota can contaminate patient urine during a UTI. (Yamamoto et al., 1997; Jantunen et al., 2001; Paalanne et al., 2018). Microbiome sequencing of human feces revealed that high abundance of either *Escherichia* or *Enterococcus* is a risk factor for bacteriuria and symptomatic UTIs (Magruder et al., 2019). While previous evidence suggested that women with *E.* coli UTIs have a different strains in the urine and gut; Magruder and colleagues observed that E. coli strains are closely related, further supporting the hypothesis of a gut microbiota-UTI axis (Bahadori et al., 2019; Magruder et al., 2019). Interestingly, a study investigating genomic diversity of UTI patients between urine and feces strains observed two major patterns: the first case being that rUTIs are caused by the same strains and the opposite, there is a rapid and complete overtake of the bladder environment by another strain (Chen et al., 2013). These studies highlight the remaining lack of clarity in developing theories to describe the origins of gut-associated UTIs. Furthermore, all present studies lack longitudinal data that would be useful in describing potential reservoirs that link gut microbiota changes before and after the onset of uncomplicated UTIs (Chen et al., 2013). With these observations in the interconnected urogenital ecosystem, we can begin to decipher ecological interactions and control mechanisms between hosts and microbes, which drive changes in microbiota composition. For example, Thänert et al. (2019) deciphered the core genomic relatedness among *E. coli*, suggesting that *E.coli* is well-adapted to transit between various environments within a host, such as between the human gut and bladder. For example, in a study investigating pairwise interactions from patient fecal-associated *E. coli* and UTI *E. coli*, the authors identified unique mutations for virulence and nutrient-uptake in addition to proteins for biofilm formation, these factors are necessary for transition between the gut and urinary tract (Nielsen et al., 2016). **Figure 2** describes the normal female urogenital microbiome during health and how gut-associated pathogens transit to both the bladder and vagina during dysbiosis.

A different aspect of the microbial reservoir route was revealed when Gottschick et al. (2017) hypothesized that the bladder and urethra have distinct microbiotas, in comparison to the vagina. It was found that abundant bacteria of the vaginal fluid from bacterial vaginosis patients were also increased in the urine. This suggests that biofilms in the urine persist for extended times as a reservoir for recurrent bacterial vaginosis infections (Gottschick et al., 2017). More so, this study highlights the need to clarify the underlying crosstalk and mechanisms of microbiota transition between the urethral and vagina. For example, it is understood that uropathogenic strains rapidly form biofilms to rapidly grow in human urine, as compared to other gut-associated *E. coli* strains (Anderson et al., 2003; Justice et al., 2004; Forsyth et al., 2018). This evidence points to a major question: is there a microbial reservoir for the initial contamination and recurrence of UTIs? If so, what mechanisms is UPEC employing to invade the urinary tract when transiting from the gastrointestinal tract?

## Antibiotic Resistance Mechanisms

The emergence of so-called “superbugs” is prevalent in global hospitals (Liu et al., 2016; Malhotra-Kumar et al., 2016; Versporten et al., 2018). The Antimicrobial Resistance Epidemiological Survey on Cystitis (ARESC) conducted a multi-national survey on various nations, in which 3018 pathogens were isolated from 4264 female patients (Schito et al., 2009). In total, 2315 (76.7%) were *E. coli* with an acquired complete resistance to ampicillin (Schito et al., 2009). These microbiota become MDR through the acquisition of genotypes through horizontal gene transfer or mutations to resist treatments (Davies and Davies, 2010; Mathers et al., 2015; Salverda et al., 2017; van der Zee et al., 2018). Increased prevalence of MDR globally is likely attributed to wide-spread and indiscriminate application of broad-spectrum antibiotics, which do not eliminate MDR pathogens (Cai et al., 2015; Goneau et al., 2015). There is a great need for the application of antibiotic stewardship surrounding UTI treatment (Rossolini et al., 2014; Bartoletti et al., 2016).

### Subinhibitory Antibiotic Treatments

**Figure 3:**
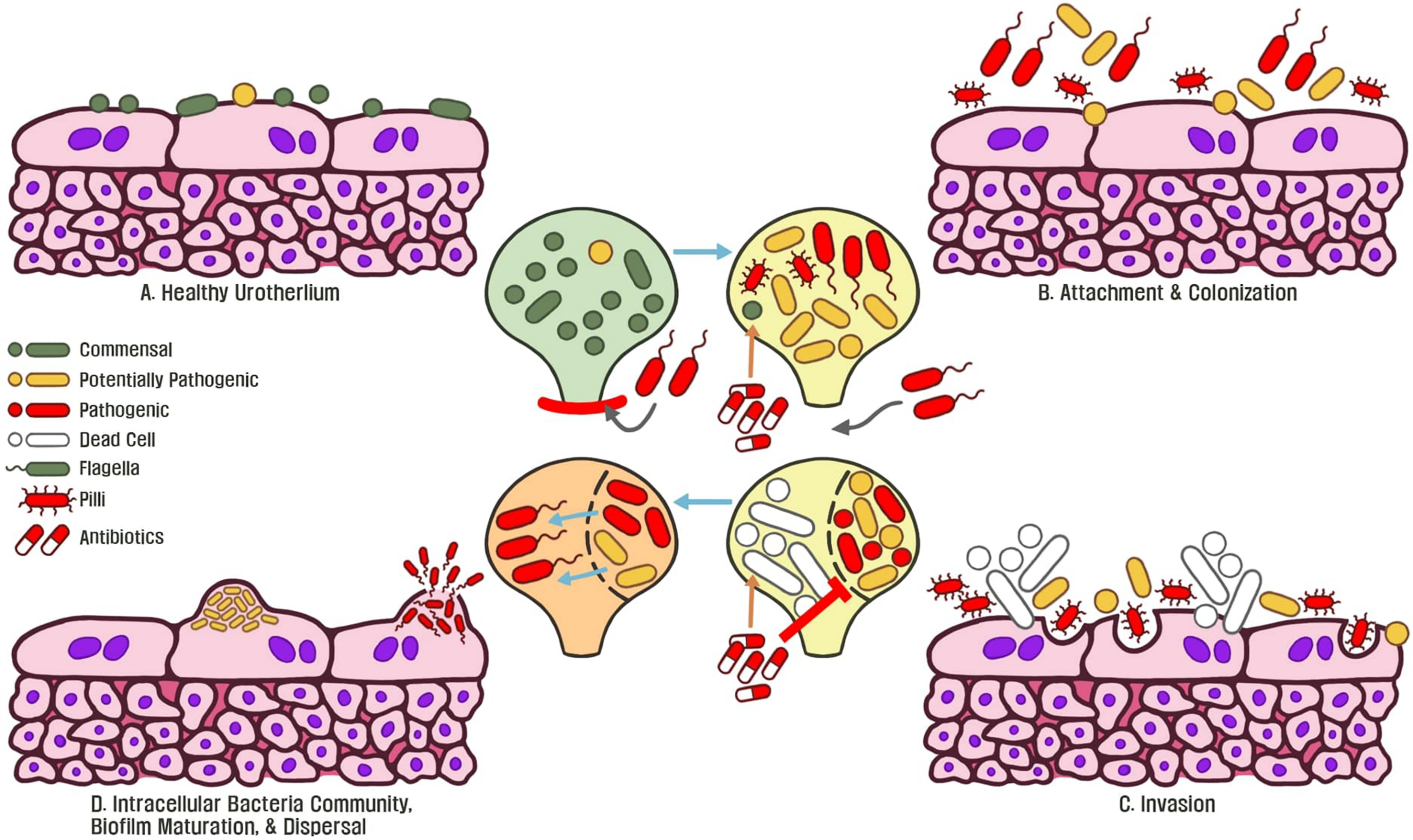
**(A)** Commensal microbiota are associated with a healthy normal human urothelium and protect the host from invasive pathogens. **(B)** An initial course of antibiotics enforces bacteria to express flagella, type 1 pili, and adhesin proteins to attach and colonize the urothelium. **(C)** Continued antibiotic exposure eliminates commensal bacteria and surviving microbiota exposed to this stressor become resistant. Invasive bacteria begin to express virulence genes to colonize and replicate within tissue. **(D)** Following replication, intracellular bacteria communities are formed and antibiotic resistant biofilms become matured. Continued exposure to stressors such as antibiotics allows for the emergence of pathogenic, MDR biofilms, which underlines recurrent chronic UTIs of the host.

In face of the emergence of horizontally-transferred MDR genes globally, UTIs are one of the most common sources for human-associated MDR pathogens due to inappropriate antibiotic usage which do not eliminate pathogens (Cullen et al., 2012; Vellinga et al., 2012; Nordstrom et al., 2013; Bartoletti et al., 2016; Salverda et al., 2017; Zilberberg et al., 2017; van der Zee et al., 2018). Through antibiotic treatment, microbiota regulate a multitude of stress response mechanisms such as mutation/modification of the genome to alter translation, produce enzymes to degrade antibiotics, and modifications in membrane permeability to counteract antibiotic stress (Dantas et al., 2008; Gniadkowski, 2008; Davies and Davies, 2010). These observations demonstrate a wide variety of mechanisms that pathogens employ to tolerate stress. Chronic usage of both low-dose and full-course antibiotics disturbs commensal microbiota, dysbiosis drives virulence and biofilm-associated resistance mechanisms by foreign pathogens, leading to recurrent infections (**Figure 3**).To map bacterial responses and adaptation, Erikson and colleagues (2017) created a database assessing transcriptome-level expression of genes coding for *E. coli* resistance and tolerance to various stressors in the environment. The authors identified that 12% of all transcript changes across all *E. coli* experiments were due to antibiotic treatment. The multiple antibiotic resistance transcriptional regulator (*marA) wa*s found to be overexpressed in the database and acts as a key-driver for MDR phenotypes in *E. coli* (Ruiz and Levy, 2010; Erikson et al., 2017). The increased expression of *marA* in uropathogenic bacteria is associated with expression of flagella proteins, biofilm production, and enhanced planktonic aggregation; all are essential for pathogenic colonization of urothelial tissue (Hadjifrangiskou and Hultgren, 2012). Furthermore, *marA* is a global regulator during antibiotic stress and activates transcription of the multidrug efflux system, which is observed in UTI patients (Schuster et al., 2017; Atac et al., 2018; Chowdhury et al., 2019; Praski Alzrigat et al., 2021).

### Polymicrobial UTI Biofilm Formation and Persistence

Biofilms are microbial communities that are formed through a complex matrix of extracellular polymeric substances, such as proteins, polysaccharides, and DNA, attached to a surface (Stewart et al., 2013). Biofilm formation is initiated when planktonic cells attach to both biotic and abiotic surfaces in an environment. Through the secretion and intake of autoinducers (small molecules or peptides), bacteria chemically interact and interpret changes in cellular density among the biofilm, in a process described as ‘quorum sensing’ (Azeredo et al., 2017). Lack of space within an environment triggers self-imposed mechanical stress for the entire biofilm community, driving biofilm formation and uptake of nutrients at the periphery of the colony (Chu et al., 2018). Cells sense both surfaces and intermingling microbiota, thus biofilm communities in tandem are able to dynamically orchestrate gene expression to produce adhesins for forming multicellular communities (Papenfort and Bassler, 2016).

Most cases of lower UTIs with UPEC infections are diagnosed as monomicrobial (Pruetpongpun et al., 2017). Specifically, UPEC strains harbor numerous virulence factors to enable survival and persistence of polymicrobial communities to persist for months against harsh host environments, thus acting as a reservoir for recurring infections in various human niches (Meier et al., 2008; Luo et al., 2012; Bjarnsholt, 2013). UPEC strains generally have a higher number of fimbrial gene clusters such as adhesins and motility complexes, such as type 1 fimbriae or pili, and flagella which greatly enhance the ability of UPEC to form biofilms (Pratt and Kolter 1998; Spurbeck et al., 2011; Luo et al., 2012; Sharma et al., 2016). In the case of the human urinary tract, the formation of polymicrobial biofilms occurs as a reaction to stressful conditions such as sub-inhibitory antibiotic exposure, to shelter bacteria from harm (Penesyan et al., 2019). Thus, biofilms are heavily regulated by environmental signaling and pathways, especially as a defensive response to antibiotics (Hoffman et al., 2005; Karatan and Watnick, 2009). For example, rapA enhances biofilm gene regulation to increase antibiotic resistance, both ymgB and yafQ initiates E. coli leads to biofilm formation under antibiotic stress (Sharma et al., 2016).

Intracellular communities of biofilms are inhabited by multiple species that are dependent on polymicrobial interactions (chemical, physical, spatial organization) (Kostakioti et al., 2013; Marchal et al., 2017) between mixed or segregated communities. Whether multispecies interactions in polymicrobial UTI biofilms generally are synergistic or competitive remains unclear. It is difficult to paint a clear overall picture of the polymicrobial ecological interactions in UTIs, especially during antagonistic interactions when exposed to antimicrobial agents (de Vos et al., 2017). However, it is understood that organisms involved in a mutualistic symbiosis will evolve towards the benefit of each other, leading to mixing of cellular populations (Marchal et al., 2017). In contrast, competitive microorganisms tend to dissociate, thus leading to layered or otherwise divided populations (Momeni et al., 2013). The mechanisms for various species interact with one another is likely within a system to support one another through cross-feeding, co-exist in neutralism, or rigorously compete in a multispecies system respectively (Bauer et al., 2017; Ferreiro et al., 2018; Giri et al., 2019). Determination of the dominating bacterium in a polymicrobial biofilm can so far only be done in a pair-wise manner, as there is insufficient information about the entire picture of polymicrobial UTIs (de Vos et al., 2017). However, analysis of multispecies communities may determine the nature of underlying host-microbiota interactions.These challenging observations of the microbial world, involving complex species interactions, are presently being interpreted through in silico modeling of microbial communities and will be discussed later (Wootton and Emmerson, 2005; Bauer et al., 2017; García-Jiménez et al., 2018).

### Animal-Associated Pathogens as a Potential Reservoir for rUTIs

Over time, recent evidence has uncovered that the infection route of both UTIs and rUTIs goes far beyond the fecal-perianal-urethra transmission route. UPEC specifically is an intermingling pathogen which switches between humans, animals, and the environment. This paradigm described as One Health requires the standardization of new studies to better identify sources of transmission by integrating an ecological and epidemiological framework that can be translated into clinical settings (Ewers et al., 2012; Singer, 2015; Davis et al., 2017). For example, zoonotic pathogens that may or may not be MDR are reintroduced as a probable environmental reservoir for rUTIs (Nordstrom et al., 2013). Preliminary studies suggesting a potential link between animals, E. coli, in which women who consume chicken or pork were at a 3.2 and 3.7 greater risk respectively, to acquire MDR UTIs, suggesting that dietary habits are an important risk factor for E. coli associated UTIs (Shooter et al., 1970; Bettelheim et al., 1974; Linton et al., 1977; Manges et al., 2007; Vincent et al., 2010; Nordstrom et al., 2013). Furthermore, there is evidence of a sharing of E. coli and K. pneumoniae between humans and companion animals (Stenske et al., 2009; Harada et al., 2012; Naziri et al., 2016; Wang et al., 2018; Marques et al., 2019). Zoonotic gut-associated E. coli presents a risk to humans by transferring from farm or companion animals, through the environment, into humans is a significant concern with regards to MDR pathogens (Cantas and Suer, 2014; Pomba et al., 2017; Bourne et al., 2019).

Recently, the first MDR E. coli classified as a superbug on US soil acquired the mobilized colistin resistance (MCR-1) gene from a commercial hog farm and manifested itself as a clinical UTI in a human patient (McGann et al., 2016). The MCR-1 gene is readily transferred through horizontal gene transfer and translates to a resistance against colistin, a last-resort antibiotic (van der Zee et al., 2018; Wang et al., 2018; He et al., 2019). MCR-1 was observed when profiling one of the most common MDR UTI strains: ST131 (Hasman et al., 2015). The authors assessed 1,923 meat and 1,188 human clinical isolates and observed that 1.3% and 15.3% strains respectively were identified as ST131. Specifically, nearly all meat isolates of ST131 were from the H22 lineage, which corresponds with fimH22 which is involved in the expression of adhesion and host invasion genes (Hasman et al., 2015; Liu et al., 2018).Additionally, a recent study of MCR-1 was found to be wide-spread in companion animals (Wang et al., 2018). Shockingly, evidence is mounting towards the potential that microbial strains isolated from the feces of animals may be directly linked to human UTIs by identifying shared strains between both species and feces or urine (Johnson et al., 2008; Osugui et al., 2014; Marques et al., 2019).

These studies illustrate a rapidly evolving story of MDR pathogens in which the ever-increasing emergence of UTIs that are associated with the interactions of contaminated animals, presenting a significant threat to public health in both communities and clinical settings. However, the correlation between farm or companion animals should be interpreted with caution. Identifying similarities between human/animal isolates does not include the transmission of microbiota and the routes that are employed to cause disease in humans and therefore requires an integrated One Health approach to better understand the issue (Ewers et al., 2012; Singer, 2015; Davis et al., 2017).

## Host-Microbial Interactions Leading to UTI

Urine is the primary source of information for identifying UTIs in patients. Analytical chemistry techniques use urine to detect biomarkers, such as excreted metabolites from pathogens (Smart et al., 2019). Specifically, urine is a waste product from various metabolic end-points of secondary metabolism whose concentration is determined by an individual’s diet, lifestyle, and a variety of other environmental factors (Bouatra et al., 2013; Playdon et al., 2016; Tang, 2017). With regards to sex, the urine of females generally contains greater quantities of citrate, but not as much calcium or oxalate as males (Ipe et al., 2016). These metabolic differences likely favors a cohort of specific microbes which thrive in these niches. A large-scale multi-experimental mass spectrometry study of human urine samples was conducted to create the database of “The Human Urine Metabolome” to identify 2651 different metabolites and unique ionic species in healthy human urine (Bouatra et al., 2013). Overall, this study preliminary assesses the diversity of the urine metabolome in healthy humans and can be useful to predict metabolites which predisposes individuals to UTIs. However, there is a lack of data detailing the metabolic state of urine in various pathophysiologies and lack of knowledge regarding biomarkers that may predispose patients to various infection outcomes.

### Host Metabolome & Bacteria of the Urogenital System

Urine is a hostile environment for most bacterial species due to a pH range of 5.5 to 7, with an average of 6.2 (Rose et al., 2015). This biofluid is a patient-specific excretion containing a metabolomic profile, strongly dependent on health or disease (Beger et al., 2016). Advances in quantitative mass spectrometry have enabled the identification of specific metabolites as biomarkers of infections or inflammation. For example, a study identified unique biomarkers of UPEC-specific UTIs by identifying an increased ratio of acetic acid to creatinine and trimethylamine concentration in urine (Lam et al., 2014). While traditional methods such as a urine dipstick urinalysis have a variable sensitivity of 68 to 88% across different patient groups, employment of mass spectrometry was found to have a 92% true positive predictive value to diagnose urinary tract infections (Devillé et al., 2004); Lam et al., 2014). (**Table 2** illustrates directional changes in microbial abundances of the urinary tract associated with disturbances in host-metabolism. Microbiota adapt and uptake various energy sources from the host diet, likely leading to modifications of urine composition and metabolic profile (David et al., 2014; Playdon et al., 2016; Thiele et al., 2020). Generally, high quantities of glucose in the urine tends to favor bacterial growth and eventually a UTI (Wilke et al., 2015). For example *E. faecalis* is able to grow in urine containing greater concentrations of glucose and has enhanced recurrent biofilm formation potential, primarily in diabetic patients (Pillai et al., 2004). Such environments exacerbate the development of pathogenesis in patients who are affected by metabolic disorders.

**Table 2:** Microbial changes linked to host metabolic dysregulation.

State of dysbiosis	Metabolic Change	Microbiota Change	Citation
Follicular fluid during IVF	↑ Estradiol & progesterone	↑ Lactobacillus gassen, L. crispatus, L. jensenii	Pelzer et al., 2012
Kidney Transplant	↑ Folate metabolism	↑ E. coli & E. faecalis	Rani et al., 2017
N/A	↑ Levels of free glycogen	↑ Lactobacillus spp.	Mirmonsef et al., 2014
Post-Menopausal (Symptomatic)	↓ Estrogen & low glycogen	↓ Lactobacillus spp.	Muhleisen and Herbst-Kralovetz, 2016
Premenopausal	↑ Estrogen & glycogen	↑ Lactobacillus spp.	Muhleisen and Herbst-Kralovetz, 2016
Renal Tubular Acidosis	Hypercalciuria	↑ E. coli	Jamshidian et al., 2018
Type Two Diabetes Mellitus	↑ Fasting Glucose & hyperlipidemia	↑ Prevotella, Lactobacillus, and Shuttleworthia	Liu et al., 2016
Urinary Tract Infections	↑ Acetic Acid & trimethylamine	↑ E. coli	Lam et al., 2014
Urinary Tract Infections	↑ Ethanolamine	↑ E. coli	Sintsova et al., 2018
Urinary Tract Infections	↑ Acetate & Creatinine	↑ E. coli	Grochocki et al., 2017
Urosepsis	↑ Procalcitonin/Albumin ratio	↑ Increased E. coli	Luo et al., 2018

N/A, Not Applicable.

Hormonal changes that impact specific host metabolic functions play a major role in the onset of female urogenital infections. Menopause influences metabolite availability and may increase the risk of UTIs (Mody and Juthani-Mehta, 2014). Menopausal women typically have lower levels of available estrogen, thereby lowering the level of glycogen within vaginal fluids (Arnold et al., 2016). This change in the metabolic composition of vaginal fluids, leads to substantial differences in substrates that microbiota employ to attach to the vaginal epithelium (Chan et al., 1984). Therefore menopausal individuals with low estradiol levels tend to have an increased risk for rUTIs (Mody and Juthani-Mehta, 2014). In contrast, increased exposure to estrogen modulates urothelium growth and differentiation to supplement epithelial defense mechanisms against UPEC invasion and UTI recurrence (Lüthje et al., 2013; Mody and Juthani-Mehra, 2014). Clinical observations found that the application of low-doses of estriol (0.03 mg) with probiotic *Lactobacillus acidophilus* can restore vaginal microflora (Donders et al., 2010). This therapy was demonstrated to restore normal vaginal physiology and mitigated bacteria linked to bacterial vaginosis (O’Hanlon et al., 2011; Mueck et al., 2018). In a recent review of non-antibiotic treatments for the treatment of UTIs in postmenopausal women, the authors suggested that the application of topical estrogen not only normalizes vaginal microbial composition, but also represent a non-antimicrobial therapy for the prevention and treatment of UTIs (Caretto et al., 2017).

### Impact of Dietary Transition Metals on UTI Pathophysiology

Metals and their ions play major roles in human health and disease through electron exchange or redox reactions, thereby promoting cellular stress through reactive oxygen species. Chronic dietary exposure to metals in the food-chain has the potential to negatively impact human health, leading to a variety of nephron- and urinary-tract associated diseases (Zhao et al., 2017; Xu et al., 2018; Yen et al., 2018). Excessive exposure to dietary metals eventually reach the kidneys and bladder causing pathologies; heavy metals are also taken up by various microbiota to incite host infection (Subashchandrabose et al., 2014; Habibi et al., 2017; Hyre et al., 2017; Zhang et al. 2019). Therefore, interpretation of rUTIs must be updated to include the possibility that metal exposure from inhalation, diet, and cosmetics are potential risk factors for UTIs onset and recurrence. On the other hand, mitigation of urinary levels of heavy metals may prove to be an alternative treatment to rUTIs by depriving pathogens use of essential metals or nutrients in comparison to antibiotic usage (Subashchandrabose and Mobley, 2015; Bauckman et al., 2019).

Free iron (Fe) is necessary for most biological processes such as cellular respiration, DNA replication, and oxygen transport *via* hemoglobin. Therefore, there is an intense competition between the host and pathogens for Fe, Fe deprivation from uropathogens may be a primary mechanism to mitigate virulence (Subashchandrabose and Mobley, 2015; Bauckman et al., 2019). For *E. coli* pathogenesis, invasion and disruption of the host urothelium to increase the availability of free Fe^2+^/Fe^3+^ is essential to initiate and maintain virulence (Gao et al., 2012). This occurs through the promotion of the UPEC toxin 𝛂-hemolysin, which ruptures and degrades host membranes to promote hemoglobin release, thereby promoting bacterial growth and virulence (Dhakal and Mulvey, 2012). UPEC opportunistically adapts to Fe abundant conditions by increasing the expression of siderophores, to scavenge environmental metals(*ireA, irp-2, iucC)* (Zhao et al., 2009; Shields-Cutler et al., 2015 Additionally, the induction of evolved virulence genes (*chuA, fepA, fyuA*, *iroN, iucA, iutA, and sitA)* are required for Fe uptake and transport systems to incite UPEC virulence mechanisms (Subashchandrabose and Mobley, 2015; Khasheii et al., 2016; Habibi et al., 2017; Tang et al., 2018). Siderophore biosynthesis and Fe acquisition from the host-environment allows UPEC to promote asymptomatic growth by overexpressing genes for growth, fitness, and colonization (Watts et al., 2012). Uptake and use of metals by uropathogens enforces an environmental niche for invasive microbiota to resist natural and artificial stressors, thus leading to persistent host infections (Hancock et al., 2008; Subashchandrabose and Mobley, 2015; Ipe et al., 2016). During UPEC-associated UTIs, Cu levels in the urine are elevated and play an underappreciated role in UTI pathophysiology (Subashchandrabose et al., 2014). Bacterial Cu-associated virulence is not as common as Fe-associated virulence in UTIs, though it is still observed in numerous organisms. The micronutrient Cu is necessary for a variety of aerobic organisms such as bacteria, fungi, plants, and animals by supporting metabolic processes through the maintenance of proteins and metalloenzymes; conversely, the interaction of Cu ions and free oxygen radicals can damage proteins (Festa and Thiele, 2011). Hyre et al. (2017) demonstrated that the gain or loss of electrons of Cu through oxidation-reduction reactions constitutes an active host response mechanism to infections with UPEC, *Klebsiella pneumoniae*, and *Proteus mirabilis*. Ceruloplasmin, a transport protein for Cu, serves as a molecular source for Cu in the urine (Hyre et al., 2017). This molecular mechanism can be observed in clinical patients who are affected by Menkes disease, a lethal hereditary disorder of Cu metabolism leading to Cu deficiency (Tümer and Moller, 2010). Consequently, those with this Cu deficiency are especially prone to developing rUTIs. (Kim et al., 2019). In contrast, recent studies demonstrate that host-mediated mobilization of Cu into the urinary tract during UTIs occurs, this illustrates a potential novel approach to reduce bacteria which rely on Cu-virulence within the urinary tract (Hyre et al., 2017). **Table 3** describes uropathogenic mechanisms of siderophores and other transport mechanisms to acquire free metals from hosts, to elicit behaviors to colonize the urothelium.

**Table 3:** Uropathogenic uptake of various metals to incite virulence in host urothelium.

Metal	Pathogen	Gene Name (s)	Function	Citation
Cu II	UPEC	Ybt	Pathogenic siderophore	Chaturvedi et al., 2012
Cu 1+/2+	UPEC	cusC	Cu resistance & virulence mechanisms	Subashchandrabose and Mobley, 2015
Fe	UPEC	Iha	Fe siderophore uptake and virulence of bladder and kidney	Léveillé et al., 2006
Fe	UPEC	FyuA	Fe-Ybt siderophore uptake & increased virulence through biofilm in bladder	Hancock et al., 2008; Brumbaugh et al., 2013
Fe (Haem)	UPEC	ChuA	Heme receptor for iron transport and kidney invasion during UTI	Hagan and Mobley, 2009
Fe	UPEC	fepA, iroN*, iutA*, fyuA, chuA, hma, sitA	Iron uptake and metal transport*	Subashchandrabose and Mobley, 2015
Ni2+ &	UPEC	nikA	Nickel acquisition & urofitness	Subashchandrabose et al., 2014
Ni(II)	UPEC	Ybt	Metal acquisition, unknown purpose	Robinson et al., 2018
Zn2+	UPEC	ZnuACB & ZupT	Uropathogenic fitness & metal transport	Sabri et al., 2009
Ni	Uropathogenic S. aureus	NixA, NikA	Urinary tract colonization and increased fitness	Hiron et al., 2010
Ni & Co	Uropathogenic S. aureus	Cnt (Opp1)	Colonization of bladder & kidneys during UTI	Remmy et al., 2013

*Both the IutA and IroN have been observed to lead to bladder colonization.

## Exploring the Urinary Tract Environment With Systems Biological Approaches

The following represents recent advances in next-generation models and methods to examine UTI pathomechanisms. These methods reach endpoints that cannot be typically reached through traditional *in vivo* or *in vitro* models. To understand the underlying environmental mechanisms which control host invasion, these methods are useful to develop novel treatment strategies for rUTIs. To achieve precision treatment options for UTIs, the application of next-generation sequencing, genome-scale metabolic modeling, and the development of novel microfluidic or stem cells technologies to mimic the bladder is needed based on patient specifics, rather than generalizations. These next-generation methodologies foreshadow a future for treating UTIs from an ecological and systemic perspective, rather than through reductionist approaches. These examples bolster the argument for a necessary adjustment towards clinical diagnosis of infections and progression of host-specific diseases.

### Transcriptomics and the Tolerome

*E. coli* are flexible microbes that thrive in various niches and environments by tuning gene expression. These modifications allow *E. coli* to rapidly adapt to stressful environments for successive colonization during environmental stressors, like antibiotic exposure, to incite host pathogenicity (Schwartz et al., 2016; Erickson et al., 2017; Zhang et al., 2017). While details of UPEC function in human urine exist, there is a lack of consistent information about UPEC acclimation to a rapidly changing environment Hagan et al., 2010; Sintsova et al., 2019). Assessing UPEC gene expression from patients becomes convoluted due to patient-specific host factors which may induce various bacterial genes, bacterial behavioral responses, and gene expression in differing host environments (Subashchandrabose et al., 2014; Schreiber et al., 2017). Furthermore, a recent study which employed UPEC strains from humans, compared pathogenesis mechanisms from humans in mice and found a similar gene expression pattern associated with metabolic machinery (Frick-Cheng et al., 2020).The complexity to assess human UPEC infections requires updating and clarifying definitions of bacterial virulence in heterogeneous human patients for optimized precision health outcomes (Mobley 2015). This aspect of biology underlies the deepening complexity of systems-oriented interaction needed to understand patient-specific disease outcomes.

Presently, the “Tolerome” describes transcriptome-level information detailing *E. coli* tolerance and resistance response to over 89 different stressful conditions (Erickson et al., 2017). Understanding the Tolerome and environmental stress response of uropathogens at a global transcriptomic level can enable a realistic understanding of the functions underpinning microbial colonization in urine. The undertaking of this study translates to 56263 events of up- or down-regulation in 5049 different genes (Erickson et al., 2017). Comparative studies of this nature can further elucidate unique bacterial signatures of microbial communities necessary for maintaining physiological equilibrium in human hosts. For example, global pathogenic transcriptional responses are potential biomarkers of infection. For example, a recent study by Sintsova and colleagues (2019) identified a novel and universal transcriptional response which controls various transcriptional regulators, thus driving rapid growth of UPEC during infection. While these studies assess *E. coli* response to various known or stable environments, investigations of rapidly changing environments is not well understood. *E. coli* response to environments is not well-characterized(i.e during dynamic disease processes and pathophysiologies), there is a need to quantify microbial communities phenotypic reactions to host-specific perturbations and interpret how these changes impact human health or disease in real-time.

Shotgun metatranscriptomics profiles the transcriptome of all present strains of a microbial community in an environment and their functions (Shakya et al., 2019). While metagenomic sequencing provides information on the assembled genome and potential genes of organisms which comprise microbial communities, it cannot provide real-time information on the functions or metabolic diversity of these communities (Mick and Sorek, 2014). More so, while metagenomics can identify active or inactive microbial members of a community, does not provide much information towards the function of a microbial community like metatranscriptomics (Brown et al., 2017; Shakya et al., 2019). While metagenomics can provdie information on all functional possibilities of the microbiome, metatranscriptomics provides a snapshot of the present infection state of the patients. Particularly, application of metatranscriptomics deciphers both function and diversity of microbial communities in responsive and non-responsive individuals. Further advances in next-generation sequencing has led to the development of scRNA sequencing of microbial communities as a novel approach to elucidate functional interactions among microbial species, mixed microbial communities, and within the host-associated microbiome (Lloyd et al., 2018; Imdahl et al., 2020; Kuchina et al., 2021). While scRNA sequencing for microbial communities is developing, metatranscriptomics is becoming readily available to identify present organisms in microbial communities from human samples in tandem with microbe-specific gene expression changes host responses (Shakya et al., 2019). Metatranscriptomics was applied to study the vaginal microbiota during bacterial vaginosis, following antibiotic treatment (Deng et al., 2018). Subjects were divided into responsive and non-responsive groups to identify potential mechanisms underlying response to the drug. The authors observed that *G. vaginalis* upregulated CRISPR-Cas genes as a stress response to mitigate DNA strand breakage from metronidazole by inducing DNA repair mechanisms. Female patients colonized by *G. vaginalis* with this unique change in gene expression were shown to be non-responsive to antibiotic treatment. Overall the observations from this study cast doubt on previous assumptions regarding microbial communities and pathogenesis progression. Metatranscriptomics for assessing the functional roles of diverse microbial strains in various environmental conditions of humans is growing in the literature (Shakya et al., 2019). But to date, metatranscriptomics has not been applied to urine or patient samples with UTIs *in vitro* or *in vivo*. Application of metranscriptomics for the bladder microbiome would clarify perspectives of not only the microbiota occupying the bladder, but also their community interactions in respect to UTI pathophysiology.

### *In Silico* Modeling of Human-Like Bladder Environments

Organisms adapt their metabolism to maintain homeostasis in ideal or adverse environments by selecting either rapid growth or resistance phenotypes (Ewald et al., 2017). Specifically, the metabolic flux of prokaryotes dynamically adjusts during growth alterations, thus optimal adaptation of protein production depends on pathway expression that constrains or allows growth (Bartl et al., 2013). Fulfillment of metabolic requirements to maintain physiological balance is a strategy employed by pathogens in rapidly changing environments to thrive or survive (Wessely et al., 2011). Depending on required response time, *E. coli* employs transcriptional or post-transcriptional mechanisms to regulate metabolic pathways for stress adaptation (Wessely et al., 2011). Identification and understanding of stress-response regulatory networks illustrates the role of environmental sensing and non-genetic changes in the emergence of disease phenotypes or MDR in pathogens. During this phase, the rapid acclimation of bacteria can be exploited to innovate treatment options for infectious human diseases (Conover et al., 2016).

By assessing the remarkable flexibility and diversity of differentially expressed genes between different stressful conditions, we can decipher UPEC adaptation towards colonization and persistence within differing human hosts (López-Maury et al., 2008). Transcriptional responses to various environmental cues are understood, less is known about UPEC response to heterogenous humans with regards to UTIs (Erickson et al., 2017). Furthermore, transcriptional changes that are host-pathogen specific may not be readily replicated in biological experiments, due to complexities when attempting to control specific environments. Thus, the application of genome-scale metabolic models for uropathogens which predicts an organisms’ transcriptional and metabolic changes when primed by environmental stresses is a promising approach to unravel the complexities pathophysiology and predict treatment strategies to prevent or intervene against rUTIs (Józefczuk et al., 2010). The mapped genomes of various wild-type and mutant *E. coli* strains have been defined and applied to predict or engineer various metabolic capabilities through reconstruction of constraint-based genome-scale models. Modeling metabolic systems has been constantly updated since the original metabolic network reconstruction of *E. coli* in 2000 (Edwards and Palsoon, 2000; Orth et al., 2011; King et al., 2015; O’Brien et al., 2015). Genome-scale metabolic modeling is useful for creating genome-wide transcriptional networks to illustrate transcriptional response to various environmental stresses. By integrating both transcriptomics and metabolomics to identify conserved responses for various environmental stressors, such as oxidative stress, acid, cold, heat, and shifting glucose to lactose in media which are necessary for the maintenance of homeostasis (Józefczuk et al., 2010; Seo et al., 2015; Du et al., 2019). Application of genome-scale metabolic models that incorporate environmental stressors provides inspiration to interpret uropathogenic transcriptomes which are associated with chronic infections.

*In silico* community metabolic models of microbiota provide valuable insights into the ecological interactions within a microbial consortia. In brief, metabolic models of microbial species are constructed based on the organism’s annotated genome sequence. Enzymes, for which predicted genes are assigned to reactions, which the enzymes can catalyze. By connecting the individual reactions to a metabolic network, *in silico* representations of the organism’s catalytic capabilities allow predictions of metabolic phenotypes, for instance environmental context-dependent growth rates, nutrient utilization, and metabolic by-products release (O’Brien et al., 2015). Models of community metabolism are constructed by connecting species-specific models and allowing the exchange of metabolites between cells as well as the competition for shared resources. To our knowledge, there are no applications of community metabolic models of the urinary tract microbiome. However, *in silico* modeling of the human gastrointestinal microbiota revealed specific metabolic processes, including fermentation and metabolic cross-feeding interactions that are altered in disease when compared to healthy controls (Bauer and Thiele, 2018; Graspeuntner et al., 2018; Aden et al., 2019; Pryor et al., 2019). The rationale behind such models is that microorganisms not only adjust their metabolism in response to abiotic stresses and chemical composition of the environment, but also depend on the presence or absence of other microbial cells in their vicinity (Klitgord and Segrè, 2011).

In systems medicine, these models are applicable to generate hypotheses of factors that promote ecosystem colonization with a potential pathogen. Thereby providing potential treatment options, as well as prevention strategies against resistant pathogens. Thus with the growing appreciation of the role of the urinary tract microbiome in rUTIs, *in silico* models of community metabolism can expand clinical understanding of rUTI pathophysiology and foster the development of novel therapeutic strategies to target the urobiome specifically.

### *In Vitro* Modeling of Human-Like Bladder Environments

For designing an *in vitro* model reflective of human rUTIs that yields meaningful data, mimicking human conditions realistically is necessary. Identification of a relevant urine medium, characterization of the physical bladder environment, and understanding the patient-specific urobiome function is required. Standard artificial urine medium that was first detailed in 1961 for UTI modeling is based on the 17 most prevalent urine substances (Altman, 1961; Brooks and Keevil, 1997). However, Boutra and colleagues identified more than 2600 unique metabolite species in healthy urine by aggregating urine from multiple donors to compensate for missing metabolites (Boutra et al., 2013). Consequently, this approach to urine modeling further complicates reproducibility due to urine metabolite composition and variability. To our knowledge, there is a deficiency of studies detailing uropathogen adaptation to various environmental conditions of healthy urine or during pathophysiological changes over time.

Due to the advancing need to model and replicate human disease outside of human systems and away from animals in a translational way towards precision medicine, a plethora of methods have been developed to interpret complex human diseases *in vitro*. Generally, preliminary animal models have provided the investigative foundation to understand human systems, but consistently fail to replicate and provide specific insights into the complexity of the human system, let alone the intricacy of the human microbiome over a lifetime. The nature of precision medicine requires that advances move away from the testing of *in vivo* models which do not reflect human health. There is a rapid drive to develop *in vitro* models which accurately mimic human systems.For instance, a flow chamber culture system was developed to model human bladder infection in real-time to observe the phenotypic changes. These dynamic UPEC changes demonstrated a switching between filamentous or rod-shape forms to facilitate secondary infections, which could not be observed in standard *in vivo* or static *in vitro* models (Andersen et al., 2010; Andersen et al., 2012). (). In a follow-up study of the same cultured flow chamber system, *E. coli was allowed to* flow through a microfluidic system, in comparison to static growth (Stærk et al., 2015). A key difference was flowing E. coli expressed the adhesive type-1 fimbriae, as an essential regulator for bladder cell adhesion,colonization, biofilm formation, and eventually persistence in urothelium (Stærk et al., 2015). Recently, a novel microfluidic biosensor for monitoring and measuring electrical fluctuations caused by mechanical movements of single bacteria was developed (Kara et al., 2018).This model can uniquely employ patient-derived urine to study UPEC movement and susceptibility to various host-derived factors in real-time (Kara et al., 2018). These works illustrate the benefits of engineered microfluidic-based systems for UPEC time-course studies, in comparison to static models which do not accurately reflect infection in the human host.

Stem cells used to create human-like systems to model disease pathophysiology, towards drug discovery, and toxicity testing by developing cell lines relevant to humans for precision medicine (Singh et al., 2015). Advances in UTI modeling is becoming closer to reality as researchers design *in vitro* approaches to employ induced pluripotent stem cells from donors to differentiate stem cells into various cell types for creating organ-like structures. One patient-specific method surgically collects human urothelium from donors to create organoid cultures, relevant to a patient-specific bladder and urinary tract (Varley and Southgate, 2011). More recently, Horsley et al. (2018) presented a new method for organoid development which relies on urine for characterizing various infection types. This novel organoid model employs a human-based urothelium model as a platform to study the relationship between hosts and pathogens in a variety of environments (Horsley et al., 2018). Overall, these advances describe the rapid movement towards redefining human UTIs by transplanting patients into a laboratory setting, and vice-versa. In principle, this paves a path towards studying the interactions of host-specific cells with microbiota in application to personalized medicine.

## Conclusion

This review challenges previous interpretations of UTI treatment and diagnosis to discuss UTIs as a host-centric disease, requiring a holistic approach. There is an overarching need to understand the uncomplicated UTIs as a dynamic system, complicated by host-associated environments and dysbiotic microbial communities. Integrating the ecological interactions between the host and microbial factors is necessary to progress rUTI diagnosis and therapy in the age of antimicrobial resistance. This integration includes observations of the female bladder microbiome in health and disease, host metabolic dysregulation, and dietary contaminants such as antibiotics or transition metals. This information can be processed through the combination of transcriptomics, *in silico* metabolic modeling or systems biology methods, and culturing induced pluripotent stem cells with microfluidic systems which are representative of the host. Overall, the presentation of the ‘uncomplicated environment of UTIs’ perspective can become an essential framework to further assess the effects of UTIs and their treatments on entire patients, rather than only the urinary tract. Thus advancing systems biology applications and precision medicine practices towards understanding UPEC pathology and other urogenital colonizing pathogens is ultimately necessary to effectively treat patients. The broad aspect of systems medicine in tandem with omics-based analysis and *in vitro* modeling can pave the way towards a mechanistic understanding of evidence-based diagnosis for rUTIs. To date, there are no systems medicine works on UTIs. Systems medicine is evolving more rapidly as available data becomes available and is translated into patient-specific models of biological systems. In particular, UTIs are not only one of the most common types of human infections but it is also poorly understood as a complicated system. That is why presently, scientists, biomedical engineers, physicians, and bioinformaticians have the best chance together, for combinatorial efforts towards employing precision treatments for rUTIs while maximizing benefits to patients.

## Author Contributions

JJ-S drafted the manuscript. TK, HR, SG, ML, MC, SW, JR, JM-J, and CK provided extensive input and materials into the support of this work. JJ-S and TK drafted and refined the figures. All authors contributed to the article and approved the submitted version.

## Funding

We acknowledge financial support from the Land Schleswig-Holstein within the funding program Open Access Publication Fund”.

## Conflict of Interest

The authors declare that the research was conducted in the absence of any commercial or financial relationships that could be construed as a potential conflict of interest.
